# Serotonin and dopamine receptors in motivational and cognitive disturbances of schizophrenia

**DOI:** 10.3389/fnins.2014.00395

**Published:** 2014-12-04

**Authors:** Tomiki Sumiyoshi, Hiroshi Kunugi, Kazuyuki Nakagome

**Affiliations:** ^1^Department of Clinical Research Promotion, National Center Hospital, National Center of Neurology and PsychiatryTokyo, Japan; ^2^Department of Mental Disorder Research, National Institute of Neuroscience, National Center of Neurology and PsychiatryTokyo, Japan; ^3^National Center Hospital, National Center of Neurology and PsychiatryTokyo, Japan

**Keywords:** serotonin, 5-HT receptors, motivation, cognition, schizophrenia, dopamine, negative symptoms, psychosis

## Abstract

Negative symptoms (e.g., decreased spontaneity, social withdrawal, blunt affect) and disturbances of cognitive function (e.g., several types of memory, attention, processing speed, executive function, fluency) provide a major determinant of long-term outcome in patients with schizophrenia. Specifically, motivation deficits, a type of negative symptoms, have been attracting interest as (1) a moderator of cognitive performance in schizophrenia and related disorders, and (2) a modulating factor of cognitive enhancers/remediation. These considerations suggest the need to clarify neurobiological substrates regulating motivation. Genetic studies indicate a role for the monoamine systems in motivation and key cognitive domains. For example, polymorphism of genes encoding catecholamine-O-methyltransferase, an enzyme catabolizing dopamine (DA), affects performance on tests of working memory and executive function in a phenotype (schizophrenia vs. healthy controls)-dependent fashion. On the other hand, motivation to maximize rewards has been shown to be influenced by other genes encoding DA-related substrates, such as DARPP-32 and DA-D_2_ receptors. Serotonin (5-HT) receptors may also play a significant role in cognitive and motivational disabilities in psychoses and mood disorders. For example, mutant mice over-expressing D_2_ receptors in the striatum, an animal model of schizophrenia, exhibit both decreased willingness to work for reward and up-regulation of 5-HT_2C_ receptors. Taken together, genetic predisposition related to 5-HT receptors may mediate the diversity of incentive motivation that is impaired in patients receiving biological and/or psychosocial treatments. Thus, research into genetic and neurobiological measures of motivation, in association with 5-HT receptors, is likely to facilitate intervention into patients seeking better social consequences.

## Introduction

Disturbances of mental processes, including cognitive function (e.g., several types of memory, attention, processing speed, and executive function, fluency) and motivation characterize many of the psychiatric illnesses, such as schizophrenia, mood disorders, and substance abuse (Simpson et al., 2011; Choi et al., 2014; Sumiyoshi, in press). Recently, the development of biological (e.g., pharmacotherapy and brain stimulation) and psychosocial (e.g., cognitive rehabilitation) interventions is targeting social function/adaptation as an important outcome measure (Harvey et al., 2011; Leifker et al., 2011). In this context, negative symptoms (decreased spontaneity, social withdrawal, and blunt affect) and cognitive impairment provide a major determinant of long-term outcome. Specifically, motivation deficits have been attracting interest as a moderator of (1) cognitive performance in patients with schizophrenia and related disorders, and (2) beneficial influence of cognitive enhancers/remediation (Fervaha et al., 2014; Strauss et al., 2014). These considerations suggest the need to clarify neurobiological substrates regulating motivation for improving quality of life in a rational and effective manner.

We herein present a theory/hypothesis that the research into genetic and neurobiological measures of motivation, linked to serotonin (5-HT) receptors, would facilitate treatment of patients with schizophrenia or other psychiatric illnesses.

## Motivational disturbances in schizophrenia

Schizophrenia is characterized by a range of symptoms, e.g., positive symptoms (delusions, hallucinations, thought disorders), negative symptoms, mood symptoms, and cognitive impairment. Specifically, there is a suggestion that negative symptoms can be separated into two domains; (1) a motivational dimension, consisting of avolition, anhedonia, and asociality, and (2) a diminished expressivity dimension, consisting of restricted affect and alogia (Strauss et al., 2014). There is a general consensus that motivational disturbances may overlap some (e.g., anhedonia), but not all (e.g., blunt affect, alogia) aspects of negative symptoms. The former dimension has been considered to be of greater importance in terms of functional outcome, quality of life, and recovery from the disease (Strauss et al., 2014). Whether other aspects of symptomatology of schizophrenia (e.g., mood symptoms) may substantially affect motivation in patients or vulnerable people remains to be determined (Schlosser et al., 2014).

## Dopamine (DA) systems governing motivation and cognition

The neural basis for intrinsic motivation has been an issue of extensive research. For example, activity of the anterior striatum and prefrontal cortex (PFC), measured by the functional MRI, has been shown to be associated with intrinsic motivation (Murayama et al., 2010). This line of anatomical evidence is consistent with genetic studies indicating a role for the monoamine systems in cognition and motivation, as discussed below.

The Val158Met polymorphism of the genes encoding catecholamine-O-methyltransferase (COMT), an enzyme catabolizing DA, affects performance on tests of working memory and executive function in a phenotype (schizophrenia vs. healthy controls)-dependent fashion (Egan et al., 2001). Thus, individuals with the val/val carriers in *COMT* show greater efficacy of the enzyme, leading to decreased DA levels in the PFC. The enzyme has also been suggested to mediate uncertainty-based exploration that is linked to DA levels in the PFC. For example, individuals with at least one met-allele show enhanced exploration compared to those with val/val genotype (Frank et al., 2007).

On the other hand, motivation to maximize rewards has been shown to be influenced by other DA-related genes expressed in the striatum/nucleus accumbens (NAc). Specifically, reward learning and negative reward avoidance are affected by genotypes of a polymorphism (rs907094. A/G) of the gene encoding DARPP-32 (a protein required for synaptic plasticity and reward learning mediated by DA-D_1_ receptors) and the D_2_ receptor (related to avoidance of negative outcomes), respectively (Frank et al., 2007; Klein et al., 2007). Thus, individuals with T/T genotype show greater expression of mRNA for the DARPP-32 gene, leading to greater performance to maximize rewards compared to C-allele carriers (reviewed in Frank et al., 2009). Similarly, T/T carriers of genes encoding D_2_ receptors are associated with greater density of these receptors in the striatum and greater likelihood to maximize rewards (Hirvonen et al., 2004; Frank et al., 2007). A recent study (Simpson et al., 2013) reported that overexpression of D_3_ receptors, a member of the D_2_ receptor family, in the striatum selectively impaired incentive motivation, as measured by an operant task.

The mechanisms by which DA receptors govern motivation and cognitive functions may involve timing perception. For example, genetically-engineered mice overexpressing D2 receptors in the striatum have been shown to elicit impaired working memory, behavioral flexibility and sensorimotor gating, i.e., behavioral abnormalities reminiscent of schizophrenia (Kellendonk et al., 2006). These model animals also demonstrate reduced motivation, as well as alteration of interval timing organization, as measured by the operant timing task (Drew et al., 2007). Further studies indicate that the impaired timing in these mutant mice mediates the ability of decreased motivation to worsen cognitive functions, including working memory and attention (Ward et al., 2009). These lines of evidence suggest a strategy for the intervention into motivational disturbances, in terms of biological and/or tailor-made treatments.

**Figure 1:**
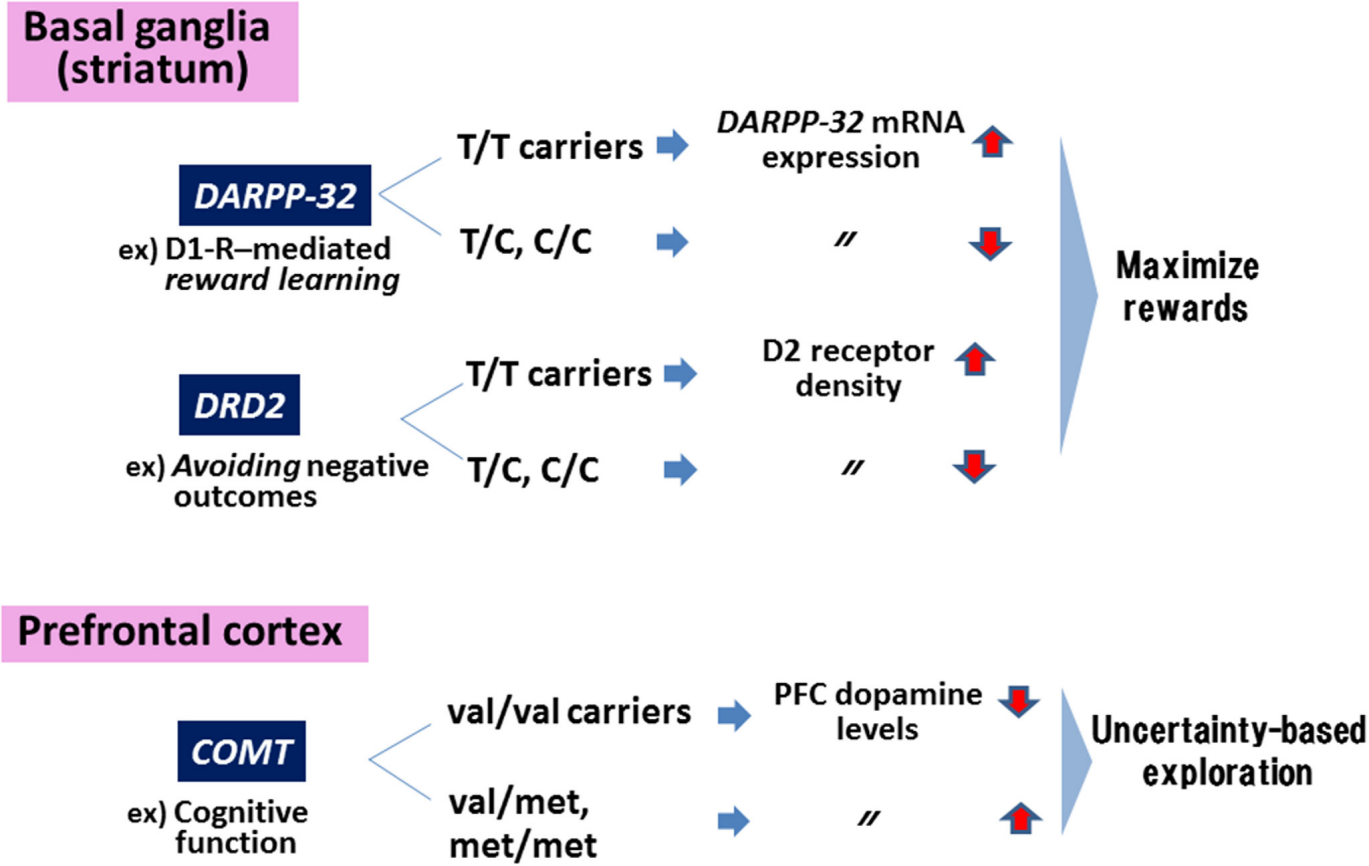
**Genes in the dopaminergic motivational system**. Polymorphisms of genes encoding *DARPP-32* and dopamine (DA)-D_2_ receptors *(DRD2)* affect behaviors to maximize rewards, while the polymorphism in *COMT* are associated with uncertainty-based exploration. Information in the Figure was extracted from Frank et al. (2009).

Figure 1 summarizes a concept about how genes encoding these DA-related substrates contribute to cognitive and motivational behaviors.

## 5-HT receptor subtypes in motivation-related behaviors

5-HT receptors, e.g., 5-HT_1A_, 5-HT_2A_, and 5-HT_2C_ subtypes, may also play a role in cognitive and motivational disabilities in psychoses and mood disorders (Meltzer and Massey, 2011; Newman-Tancredi and Albert, 2012; Ohno et al., 2012). For example, several antipsychotic and antidepressant drugs have been suggested to ameliorate negative symptoms and mood disturbances, partly through actions on 5-HT_1A_ and 5-HT_2A_ receptors (Newman-Tancredi and Albert, 2012; Ohno et al., 2012; Sumiyoshi et al., 2013; Sumiyoshi, 2014). Clozapine, the prototype of atypical antipsychotic drugs, which is most effective in treating negative symptoms, may act as an inverse agonist on 5-HT_2C_ receptors (Meltzer and Massey, 2011).

**Figure 2:**
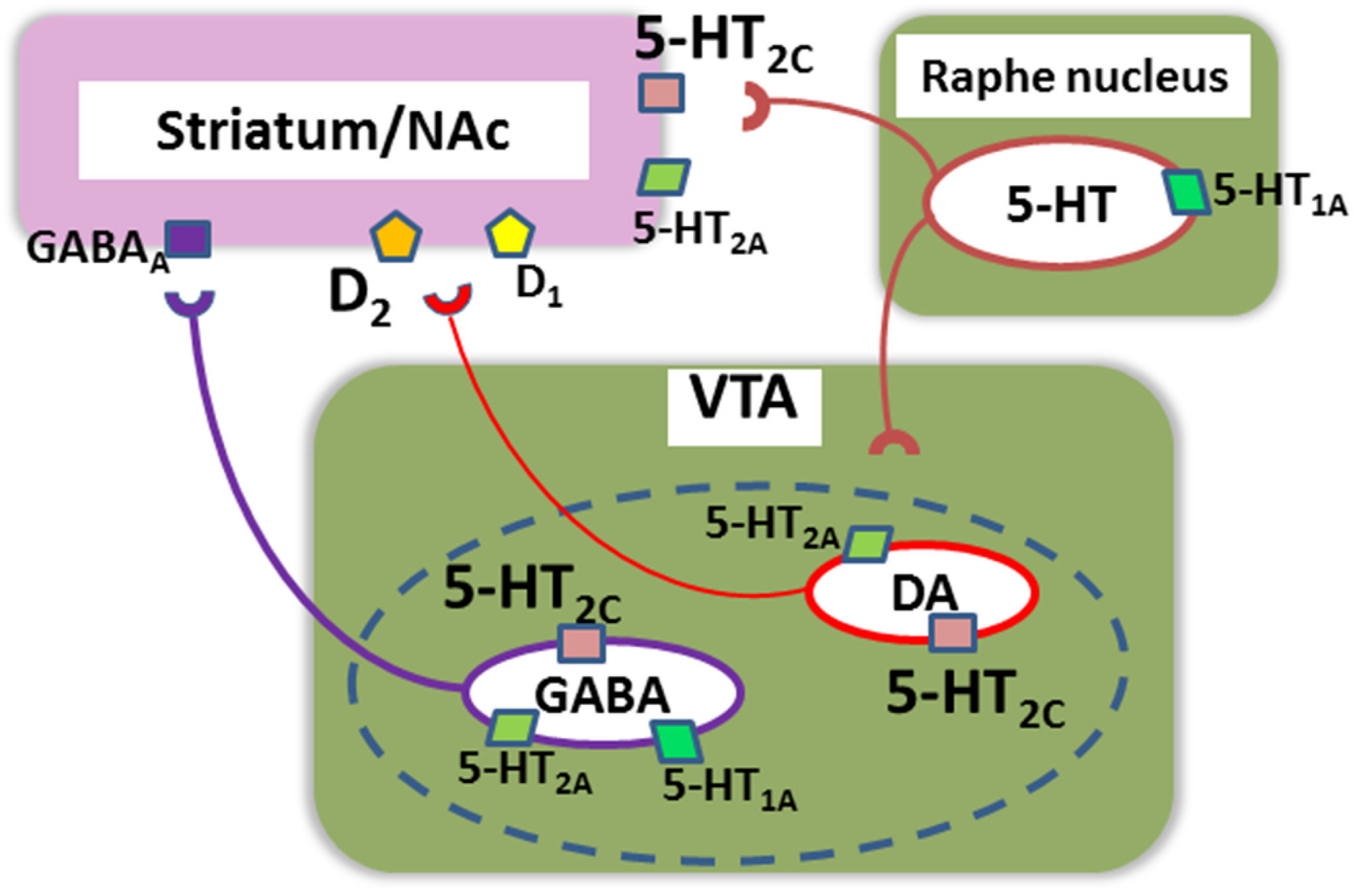
**A putative neural network mediating motivational behaviors in relation to serotonin (5-HT) receptors**. (1) Up-regulation of 5-HT_2c_ receptors in the nucleus accumbens (NAc)/striatum may be associated with a decrease in incentive motivation in mutant mice over-expressing dopamine (DA)-D_2_ receptors in the striatum, an animal model of schizophrenia (Simpson et al., 2011). SB242084, a selective *ant*agonist at these receptors, increases incentive motivation in these model mice. (2) 5-HT_2c_ receptors localized in DA and GABA neurons in the ventral tegmental area (VTA) also affect motivation by modulating transmissions to NAc, including actions on D_1_ and D_2_ receptors (Bubar et al., 2011). The dotted line indicates that a proportion of NAc-projecting VTA neurons releases both DA and GABA (Bubar et al., 2011). (3) Other 5-HT receptor subtypes, such as 5-HT_1A_ and 5-HT_2A_, may also directly or indirectly regulate this neural system of motivational behaviors.

Data from recent investigations support the contribution of 5-HT receptors to motivational behaviors. For example, mutant mice over-expressing D_2_ receptors in the striatum, exhibit both decreased willingness to work for reward and up-regulation of 5-HT_2C_ receptors (Simpson et al., 2011). Furthermore, increased D_1_, D_2_ and 5-HT_2C_ receptors co-exist in mice mis-expressing ADAR2, an RNA-editing enzyme, and these animals elicit altered expression of reward-related mRNAs in the brain (Akubuiro et al., 2013). Collectively, these observations indicate the importance of some 5-HT receptor subtypes, e.g., 5-HT_2C_ receptors, in the pathophysiology and treatment of motivational disturbances associated with psychoses (Figure 2).

The role for 5-HT_2C_ receptors in psychiatric symptoms relevant to functional outcome is also supported by observations in mice whose 5-HT-synthesizing enzyme (tryptophan hydroxyxlase-2) was genetically engineered (Del'Guidice et al., 2014). Thus, treatment with the 5-HT_2C_ agonist CP809,101 ameliorated impairments in cognitive flexibility and reversal learning in these mutant animals (Del'Guidice et al., 2014).

As noted above, up-regulation of 5-HT_2c_ receptors in the striatum may be associated with a decrease in incentive motivation (Simpson et al., 2011). Further, 5-HT_2c_ receptors localized in DA and GABA neurons in the ventral tegmental area (VTA) also have been suggested to regulate motivation by modulating transmissions to NAc (Bubar et al., 2011) (Figure 2). It should be noted that a proportion of NAc-projecting VTA neurons may release both DA and GABA (Bubar et al., 2011). Altered balance in this complicated 5-HT_2c_ receptor-associated network is postulated to cause reward-related disorders, such as schizophrenia, depression, and addiction (Bubar et al., 2011).

Other 5-HT receptor subtypes, such as 5-HT_1A_ and 5-HT_2A_receptors, may directly or indirectly influence this neural system for motivational behaviors as well. For example, 5-HT_1A_ receptor gene promotor polymorphism (rs6295, C-1019G) has been associated with treatment effects on negative symptoms of schizophrenia (Reynolds et al., 2006). Figure 2 illustrates a putative neural network mediating motivational behaviors in relation to 5-HT receptors, which, together with Figure 1 (upper part), may suggest the contribution of DA-5-HT interactions.

## Clinical perspectives and future directions

Based on the discussions so far, drugs acting on some 5-HT receptor subtypes, particularly, 5-HT_2C_ receptors, are likely to improve motivational deficits in individuals with schizophrenia. For example, SB242084, a selective *ant*agonist at 5-HT_2C_ receptors, has been shown to increase incentive motivation in mice over-expressing D_2_ receptors in the striatum, an animal model of schizophrenia (Simpson et al., 2011). By contrast, the 5-HT_2C_ receptor *ago*nist CP809,101 has been demonstrated to enhance performance on some cognitive tasks in mice with decreased 5-HT synthesis (Del'Guidice et al., 2014). These preclinical observations warrant clinical studies of the effect of agents for specific 5-HT receptor subtypes, e.g., 5-HT_2C_ receptors, on motivational and cognitive disturbances. Specifically, it is important to see if such putative pro-motivation drugs will lead to improvement of functional outcome affected by cognitive function on which such compounds might act in variable directions.

In view of a possible influence of motivation on cognitive training, it may be interesting to determine if augmentation with pro-motivation compounds, e.g., 5-HT_2C_ agents, would provide additional merits for cognitive and functional outcome in patients with schizophrenia. Also, whether genetic variations regarding 5-HT and/or DA receptors affect motivational response to treatment with existing pharmacological or psychosocial interventions deserves further study.

In summary, genetic predisposition related to 5-HT and DA receptors may mediate the diversity of incentive motivation that is impaired in patients with schizophrenia. This concept is expected to facilitate rational treatment with biological and/or psychosocial tools to improve social consequences for people with psychiatric illnesses.

### Conflict of interest statement

The authors declare that the research was conducted in the absence of any commercial or financial relationships that could be construed as a potential conflict of interest.

## References

[B1] Akubuiro A. Bridget Zimmerman M. Boles Ponto L. L. Walsh S. A. Sunderland J. McCormick L.. (2013). Hyperactive hypothalamus, motivated and non-distractible chronic overeating in ADAR2 transgenic mice. Genes Brain Behav. 12, 311–322. 10.1111/gbb.12020 23323881 PMC4589229

[B2] Bubar M. J. Stutz S. J. Cunningham K. A. (2011). 5-HT(2C) receptors localize to dopamine and GABA neurons in the rat mesoaccumbens pathway. PLoS ONE 6:e20508. 10.1371/journal.pone.0020508 21687728 PMC3110193

[B3] Choi J. Choi K. H. Felice Reddy L. Fiszdon J. M. (2014). Measuring motivation in schizophrenia: is a general state of motivation necessary for task-specific motivation? Schizophr. Res. 153, 209–213. 10.1016/j.schres.2014.01.027 24529609 PMC3962084

[B4] Del'Guidice T. Lemay F. Lemasson M. Levasseur-Moreau J. Manta S. Etievant A.. (2014). Stimulation of 5-HT2C receptors improves cognitive deficits induced by human tryptophan hydroxylase 2 loss of function mutation. Neuropsychopharmacology 39, 1125–1134. 10.1038/npp.2013.313 24196946 PMC3957106

[B4a] Drew M. R. Simpson E. H. Kellendonk C. Herzberg W. G. Lipatova O. Fairhurst S.. (2007). Transient overexpression of striatal D_2_ receptors impairs operant motivation and interval timing. J. Neurosci. 27, 7731–7739. 10.1523/JNEUROSCI.1736-07.2007 17634367 PMC6672869

[B5] Egan M. F. Goldberg T. E. Kolachana B. S. Callicott J. H. Mazzanti C. M. Straub R. E.. (2001). Effect of COMT Val108/158 Met genotype on frontal lobe function and risk for schizophrenia. Proc. Natl. Acad. Sci. U.S.A. 98, 6917–6922. 10.1073/pnas.111134598 11381111 PMC34453

[B6] Fervaha G. Agid O. Foussias G. Remington G. (2014). Effect of intrinsic motivation on cognitive performance in schizophrenia: a pilot study. Schizophr. Res. 152, 317–318. 10.1016/j.schres.2013.11.037 24333003

[B7] Frank M. J. Doll B. B. Oas-Terpstra J. Moreno F. (2009). Prefrontal and striatal dopaminergic genes predict individual differences in exploration and exploitation. Nat. Neurosci. 12, 1062–1068. 10.1038/nn.2342 19620978 PMC3062477

[B8] Frank M. J. Moustafa A. A. Haughey H. M. Curran T. Hutchison K. E. (2007). Genetic triple dissociation reveals multiple roles for dopamine in reinforcement learning. Proc. Natl. Acad. Sci. U.S.A. 104, 16311–16316. 10.1073/pnas.0706111104 17913879 PMC2042203

[B9] Harvey P. D. Raykov T. Twamley E. W. Vella L. Heaton R. K. Patterson T. L. (2011). Validating the measurement of real-world functional outcomes: phase I results of the VALERO study. Am. J. Psychiatry 168, 1195–1201. 10.1176/appi.ajp.2011.10121723 21572166 PMC3670945

[B10] Hirvonen M. Laakso A. Nagren K. Rinne J. O. Pohjalainen T. Hietala J. (2004). C957T polymorphism of the dopamine D2 receptor (DRD2) gene affects striatal DRD2 availability *in vivo*. Mol. Psychiatry 9, 1060–1061. 10.1038/sj.mp.4001561 15278099

[B10a] Kellendonk C. Simpson E. H. Polan H. J. Malleret G. Vronskaya S. Winger V.. (2006). Transient and selective overexpression of D_2_ receptors in the striatum causes persistent abnormalities in prefrontal cortex functioning. Neuron 16, 603–615. 10.1016/j.neuron.2006.01.023 16476668

[B11] Klein T. A. Neumann J. Reuter M. Hennig J. Von Cramon D. Y. Ullsperger M. (2007). Genetically determined differences in learning from errors. Science 318, 1642–1645. 10.1126/science.1145044 18063800

[B12] Leifker F. R. Patterson T. L. Heaton R. K. Harvey P. D. (2011). Validating measures of real-world outcome: the results of the VALERO expert survey and RAND panel. Schizophr. Bull. 37, 334–343. 10.1093/schbul/sbp044 19525354 PMC3044614

[B13] Meltzer H. Y. Massey B. W. (2011). The role of serotonin receptors in the action of atypical antipsychotic drugs. Curr. Opin. Pharmacol. 11, 59–67. 10.1016/j.coph.2011.02.007 21420906

[B14] Murayama K. Matsumoto M. Izuma K. Matsumoto K. (2010). Neural basis of the undermining effect of monetary reward on intrinsic motivation. Proc. Natl. Acad. Sci. U.S.A. 107, 20911–20916. 10.1073/pnas.1013305107 21078974 PMC3000299

[B15] Newman-Tancredi A. Albert P. R. (2012). Gene polymorphism at serotonin 5-HT1A receptors: moving towards personalized medicine for psychosis and mood deficits?, in Schizophrenia Research: Recent Advances, ed Sumiyoshi T. (New York, NY: Nova Science Publishers), 337–358.

[B16] Ohno Y. Tatara A. Shimizu S. Sasa M. (2012). Management of cognitive impairments in schizophrenia: the therapeutic role of 5-HT receptors, in Schizophrenia Research: Recent Advances, ed Sumiyoshi T. (New York, NY: Nova Science Publishers), 321–335.

[B17] Reynolds G. P. Arranz B. Templeman L. A. Fertuzinhos S. San L. (2006). Effect of 5-HT1A receptor gene polymorphism on negative and depressive symptom response to antipsychotic treatment of drug-naive psychotic patients. Am. J. Psychiatry 163, 1826–1829. 10.1176/appi.ajp.163.10.1826 17012696

[B18] Schlosser D. A. Fisher M. Gard D. Fulford D. Loewy R. L. Vinogradov S. (2014). Motivational deficits in individuals at-risk for psychosis and across the course of schizophrenia. Schizophr. Res. 158, 52–57. 10.1016/j.schres.2014.06.024 25008792 PMC4152418

[B19] Simpson E. H. Kellendonk C. Ward R. D. Richards V. Lipatova O. Fairhurst S.. (2011). Pharmacologic rescue of motivational deficit in an animal model of the negative symptoms of schizophrenia. Biol. Psychiatry 69, 928–935. 10.1016/j.biopsych.2011.01.012 21414604 PMC3170714

[B20] Simpson E. H. Winiger V. Biezonski D. K. Haq I. Kandel E. R. Kellendonk C. (2013). Selective overexpression of dopamine d3 receptors in the striatum disrupts motivation but not cognition. Biol. Psychiatry 76, 823–831. 10.1016/j.biopsych.2013.11.023 24387821 PMC4047204

[B21] Strauss G. P. Waltz J. A. Gold J. M. (2014). A review of reward processing and motivational impairment in schizophrenia. Schizophr. Bull. 40 Suppl. 2, S107–S116. 10.1093/schbul/sbt197 24375459 PMC3934394

[B22] Sumiyoshi T. (in press). Cognitive impairment in schizophrenia, in Encyclopedia of Psychopharmacology, 2nd Edn., eds Stolerman I. Price L. H. (New York, NY: Springer), 1–7.

[B23] Sumiyoshi T. (2014). Serotonin(1A) receptors in the action of aripiprazole. J. Clin. Psychopharmacol. 34, 396–397. 10.1097/JCP.0000000000000135 24717255

[B24] Sumiyoshi T. Higuchi Y. Uehara T. (2013). Neural basis for the ability of atypical antipsychotic drugs to improve cognition in schizophrenia. Front. Behav. Neurosci. 7:140. 10.3389/fnbeh.2013.00140 24137114 PMC3797421

[B25] Ward R. D. Kellendonk C. Simpson E. H. Lipatova O. Drew M. R. Fairhurst S.. (2009). Impaired timing precision produced by striatal D2 receptor overexpression is mediated by cognitive and motivational deficits. Behav. Neurosci. 123, 720–730. 10.1037/a0016503 19634929 PMC2791672

